# Development of Interfacial Adhesive Property by Novel Anti-Stripping Composite between Acidic Aggregate and Asphalt

**DOI:** 10.3390/polym12020473

**Published:** 2020-02-19

**Authors:** Guohong Zhang, Jianhui Qiu, Jingzhuo Zhao, Dingbang Wei, Hui Wang

**Affiliations:** 1 Gansu Provincial Communications, Planning, Survey & Design Institute Co., Ltd., Lanzhou 730030, China; weidingbang@163.com (D.W.); huiwang1190@163.com (H.W.); 2 Department of Mechanical Engineering, Faculty of Systems Science and Technology, Akita Prefectural University, Akita 015-0055, Japan; qiu@akita-pu.ac.jp; 3 Gansu Changlong Highway Maintenance Technology Institute Co., Ltd., Lanzhou 730030, China

**Keywords:** interfaces, asphalt anti-stripping agent, thermal stability, asphalt mixtures, acidic aggregate

## Abstract

Studies on control of and preventive measures against asphalt pavement moisture damage have important economic and social significance due to the multiple damage and repair of pavements, the reasons for which include the poor interfacial adhesive ability between acidic aggregates and asphalts. Anti-stripping agent is used in order to improve the poor adhesion, and decomposition temperature is regarded as being important for lots of anti-stripping products, because they always decompose and lose their abilities under the high temperature in the mixing plant before application to the pavement. A novel anti-stripping composite, montmorillonoid/Polyamide (OMMT/PAR), which possesses excellent thermal stability performance and is effective in preventing moisture damage, especially for acidic aggregates, was prepared. Moreover, the modification mechanisms and pavement properties were also investigated with reference to the composites. The results show that OMMT/PAR was prepared successfully, improving the interfacial adhesion between the acidic aggregate and the modified asphalt. Due to the nanostructure of OMMT/PAR, the thermal stability was enhanced dramatically and the interfacial adhesion properties were also improved. Furthermore, asphalts modified with OMMT/PAR and their mixtures showed excellent properties. Finally, the moisture damage process and the mechanisms by which OMMT/PAR improves the interfacial adhesion properties are explained through adhesion mechanism analyses.

## 1. Introduction

The rapid development of the highway has received much attention in the past few years because of its perfect pavement properties, operational safety and convenience [1,2,3]. Asphalt mixtures (or concrete) have been utilized in more than 90% of completed highway constructions and homogeneous buildings. However, pavement distress happens frequently with increasing use of asphalt pavements [4,5] and can even be observed during operation times [6,7,8]. The moisture damage to asphalt pavement is a major problem that negatively affects pavement performance, due to its early appearance, the rapid speed with which damage to the pavement occurs, the enormous effect on comfort and safety during driving, and so on [9,10,11,12]. That is to say, moisture damage can significantly shorten the repair cycles and the lifetime of asphalt highway, accordingly increasing the cost of maintenance and repair [13,14]. Therefore, it is necessary to study the interfacial adhesive properties between the acidic aggregate and the asphalt [15], due to the important economic and social significances [16,17].

The most important reason for the occurrence of moisture damage is the poor interfacial adhesive property between the asphalt and the aggregate [18]. Currently, an increasing number of technologies for enhancing the interfacial bonding property are being studied, such as giving priority to alkaline aggregates, or employing anti-stripping agents (ASA) [19,20]. However, these have not been implemented, for reasons as follows. First of all, alkaline aggregates are better able to bond to asphalt than acidic ones, but some of them (e.g., limestone) are not as rigid and compactible as the acidic ones, resulting in fundamental limitations in the pavement performance of asphalt mixtures. Additionally, alkaline aggregates (e.g., basalt) are suffering from resource depletion in many areas of the world, leading to other unsatisfactory substitute mineral aggregates being employed, rather than the desired ones, which would require great transportation expenses [21,22]. Furthermore, ASA may help to improve the adhesive properties between neutral minerals and asphalt, but they are always insufficient for acidic ones, showing unsatisfactory effects on different kinds of acidic aggregates. Additionally, current anti-stripping products may lead to thermal decomposition at high temperatures in asphalt mixing plants and transportation trucks [23,24]. Therefore, they are not able to improve the interfacial bonding strength between acidic aggregates and asphalt after mixing and construction. Once the water enters the asphalt concrete, hydrogen bonding can take place on the hydrophilic surface of the aggregates, displacing asphalt from the aggregate and causing stripping of the asphalt membrane [16,25]. In actual fact, mineral aggregates are often partly composed of a mixture of neutral and acidic aggregates, and it is necessary to develop new technologies or ASA materials in order to enhance the interfacial adhesive property between acidic aggregates and asphalt.

Gao et al. [26] studied four kinds of mineral with reference to the different interfacial models between aggregates and asphalt, where the bonding strength in the neutral mineral model was determined using Van der Waals’ force, while the alkaline mineral model was determined based on electrostatic attraction. Nevertheless, acidic aggregates have not been used, although they may employ a different model. Xu et al. [27] also modeled the interface between silica and asphalt, where interfacial failure was primarily attributed to bonding failure, based on the tensile simulation carried out. Meanwhile, Yao et al. [28] focused on the bonding performances between aggregates and aging asphalts, where the aging group of carboxylic acid was able to reinforce the bonding energy. Li et al. [29] investigated the bonding properties at different temperatures and with different oxide types at the interfaces between the aggregates and asphaltene. Chen et al. [1] studied the interfacial adhesion mechanism using surface microtopography, and compared different aggregates of limestone and granite, revealing that the degree of surficial texture complexity of limestone and granite decreased after abrasion, the relative flat site increased, and the surficial texture of limestone had a greater degree of influence on the interface. Chu et al. [11] employed molecular dynamics to simulate the interfacial adhesive properties between aggregates (a-quartz and calcite) and asphalts, suggesting that the surface anisotropy of the aggregates had a significant impact on the interface. Furthermore, Wang et al. [30] simulated two kinds of models, of which one was the interface between quartz and asphalt, and the other was with quartz, water and asphalt. Predictably, water decreased the bonding strength dramatically. Additionally, Fischer et al. [31] studied the interfacial adhesive strengths between aggregates and asphalt through atomic force microscopy (AFM), where the surface roughness had a stronger influence compared with the chemical nature of the surface. 

As a result, the interfacial adhesion properties between acidic aggregates and asphalt have not yet been thoroughly reported, and the anti-stripping products that are available on the market always exhibit weaker thermal stability, leading to unsatisfactory construction. Therefore, in this study, in order to improve thermal stability and for its effective application between acidic aggregates and asphalt, a novel anti-stripping composite was prepared, and the thermal, adhesive, and pavement performances are discussed in comparison with other ASA products. 

## 2. Experiment

### 2.1. Materials

To study the interface adhesive performance between asphalt and aggregate, two brands of matrix asphalts KL-90# and SK-90# were employed, and their physical properties are shown in Table 1 and Table 2. Two other anti-stripping products were used for comparison, and are referred to as ASA-1 and ASA-2, and it is possible to purchase ASA-1 and ASA-2 on the market in different countries. Meanwhile, three kinds of acidic aggregates were chosen: granite, sandstone and quartzite. Obviously, alkaline aggregates are not considered as a sample in this work due to their better adhesive properties with asphalt. The information regarding the acidic aggregates are shown in Table 3, Table 4 and Table 5. 

### 2.2. Preparation of OMMT/PAR and MAS

**Figure 1:**
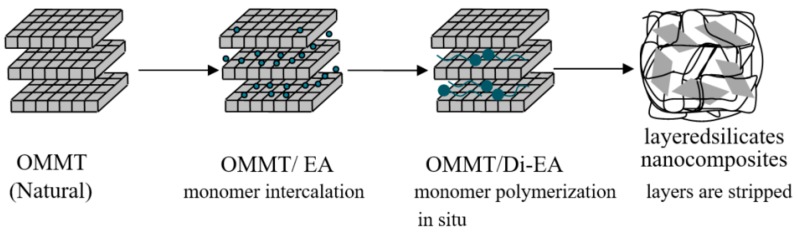
OMMT/PAR preparation of polymer intercalated by OMMT.

Anti-stripping composites were prepared according to Figure 1, where eleostearic acid (EA) and organic montmorillonoid (OMMT) were mechanically stirred under CO_2_ and gradually heated to 280 °C for 4 h, with OMMT/eleostearic dimer acid nanocomposites (OMMT/Di-EA) being obtained. Then the OMMT/Di-EA were gradually added into the diethylenetriamine (EDTA) system, which was heated to 120 °C under protection by N_2_; the system was kept under 200 °C for 2 h before EDTA was distilled under vacuum, and the resulting liquid was a polyamide nanocomposite solution. Moreover, diluent and compatilizer were added under mechanical stirring to achieve a uniform mixture when the temperature was reduced to 80 °C, and the anti-stripping layered silicate nanocomposites of OMMT/Polyamide (OMMT/PAR) were finally achieved. For the purpose of easily injecting it into the mix plant, OMMT/PAR was also diluted by glycerol in a ratio of 5:1 (OMMT/PAR: glycerol), referred to as modification anti-stripping material (MAS). PAR with no OMMT was also prepared in this work.

### 2.3. Preparation of Asphalt Mixtures 

Matrix asphalts were modified by 0.3 wt % OMMT/PAR, ASA-1, and ASA-2, respectively, where the KL-90# modified by OMMT/PAR, ASA-1 and ASA-2 was named as KL-90-OMMT/PAR, KL-90-ASA-1, and KL-90-ASA-1, respectively. Similarly, the names SK-90-OMMT/PAR, SK-90-ASA-1 and SK-90-ASA-2 were used when the asphalt was replaced by SK-90#. Matrix asphalt should always be assumed to be KL-90 unless otherwise stated.

Asphalt concrete AC 16 with a maximum grain size of 16 mm was prepared as the asphalt mixture in this work due to the particle reinforced composites and the common application on highly trafficked road pavements according to the Technical Specification for Construction of Highway Asphalt Pavements (JTG F40, TSCHAP). First of all, the modified asphalts and the acidic aggregates were heated to 150 °C and 160 °C, respectively. The hot modified asphalts and aggregates were mixed at 150 °C for 60 s, and then impacted for the revolving compaction sample with dimensions of 100 ± 2 mm in diameter and 150 ± 2.5 mm in height, and for the Marshall sample, with dimensions of 101.6 ± 0.25 mm in diameter and 63.5 ± 1.3 mm in height, according to the Standard Test Methods of Bitumen and Bituminous Mixture for Highway Engineering (JTG E20, STMBM).

### 2.4. Thermogravimetry (TG) Analysis

By using the TG-DTA thermal analyzer (Shimadzu Corp., Model DTG-60, Tokyo, Japan), the thermal performances were characterized. The test conditions were as follows: 10 mg samples were placed in an aluminum container and tested from room temperature to 800 °C at a rate of 10 °C/min.

### 2.5. Adhesive Property Test

According to the industrial standard STMBM, the adhesion properties between asphalts and aggregates are tested by utilizing the poaching method according to the rule (T0616) of STMBM. The aggregates with sizes bigger than 13.2 mm were washed and immersed into a hot asphalt sample after being heated to 105 °C and immersed for 45 s, and were then lifted out. The aggregates with the asphalt were boiled in water for 3 min after cooling in room temperature for 15 min, and the adhesion level can be marked up as having a grade of 1–5 according to the movement of hydration on the aggregate rock surface and the desquamation of the asphalt, for which grade 1 is the lowest grade and grade 5 is the highest one. To confirm the durability, adhesive tests between acidic aggregates and the modified asphalts were also carried out after aging.

### 2.6. Freeze–Thaw Splitting and Dynamic Elasticity Modulus Test of Bituminous Mixture

The freeze–thaw splitting tests divided the Marshall samples into two groups: A and B. Firstly, the A group samples were immersed in water for 15 min in a vacuum and 30 min under normal pressure; then, they were frozen at −18 °C for 16 h, and immersed in 60 °C water for 24 h. Finally, the A and B group samples were all immersed in 25 °C water for 2 h and the splitting strengths were tested, respectively. The splitting strengths of the B group were defined as the splitting strengths of these samples. Moreover, the ratio of splitting strengths of the A and B groups were defined as the splitting strength ratios (TSR) of these samples according to STMBM. The dynamic elasticity moduli of the asphalt mixtures were also evaluated according to STMBM under a temperature of 20 °C and a frequency of 10 Hz.

## 3. Results and Discussion

### 3.1. Thermo-Stability Analysis of the Anti-Stripping Composite

The thermal stability of anti-stripping composites is considered to be one of the most significant factors, due to the high temperature in the mix plants used by building contractors and asphalt suppliers. Generally speaking, the anti-stripping composite is injected into the asphalt several hours or even days before use. However, modified asphalt needs to be stored at a temperature higher than 130 °C (it may be much higher in some areas), sometimes for a number of weeks, and thus it is essential for the anti-stripping composite to hold a better thermal stability, which can help keep them effective throughout the whole mixing and compaction processes.

**Figure 2:**
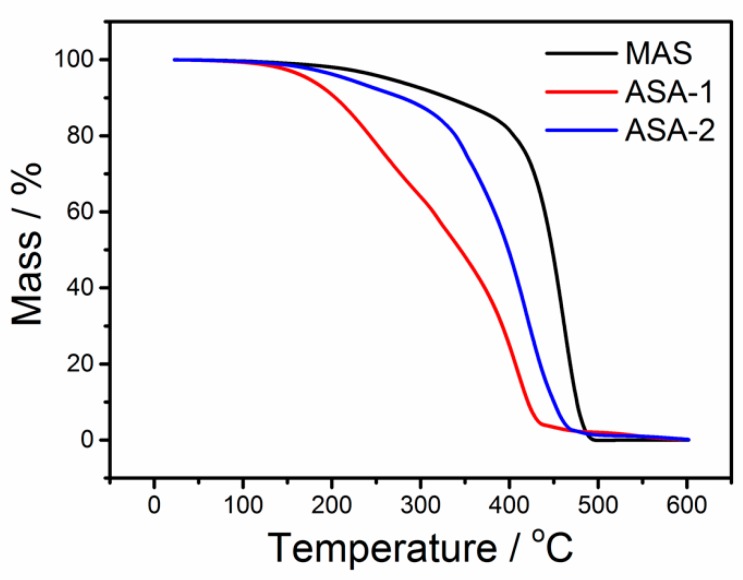
TG curves of the anti-stripping composites of MAS, ASA-1 and ASA-2.

TG curves of the three kinds of anti-stripping materials are shown in Figure 2, and the corresponding characteristic thermal data of all samples are listed in Table 6. The decomposition temperature of MAS is by 19% and 3% higher with the ones of ASA-1 and ASA-2, respectively, under 2% weight-loss conditions. Similarly, it is also 34% and 10% higher under weight-loss conditions of 5% with a decomposition temperature of 202 °C. It can be concluded that the weak thermal stability shown by ASA-1 and ASA-2 is due to their fatty amine structure, according to Introduction. MAS is a kind of ramification of acylamino, in which the anti-stripping property can be assumed to stabilize during not only mixing with aggregates in the mix plant, but also when being stored under a high temperature for a long time. The thermostable group in MAS results in high stability, leading to it having the ability to prevent decomposition.

**Figure 3:**
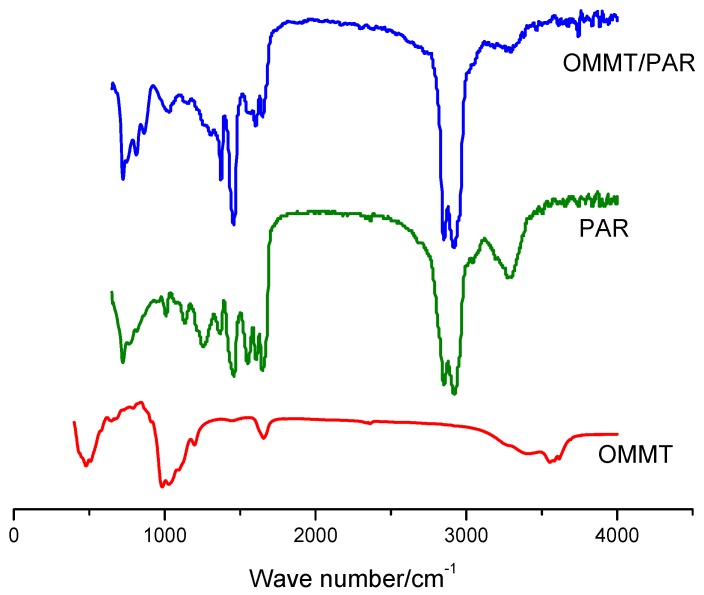
FT IR curves of OMMT, PAR and OMMT/PAR composites.

Figure 3 shows the FT IR curves for OMMT, PAR and OMMT/PAR composites, in which the absorption peak of the stretching vibration of N-H in PAR is shown at 3350 cm^−1^, and the peak of the bending vibration of C-ONH in PAR is also shown at 1250 cm^−1^, which can be considered to be typical functional groups of PAR compared with that of OMMT. Therefore, it can be concluded that the layered silicate nanocomposites of montmorillonoid were prepared successfully, based on the peak of 1250 cm^−1^ and 3350 cm^−1^ according the curve of OMMT/PAR.

**Figure 4:**
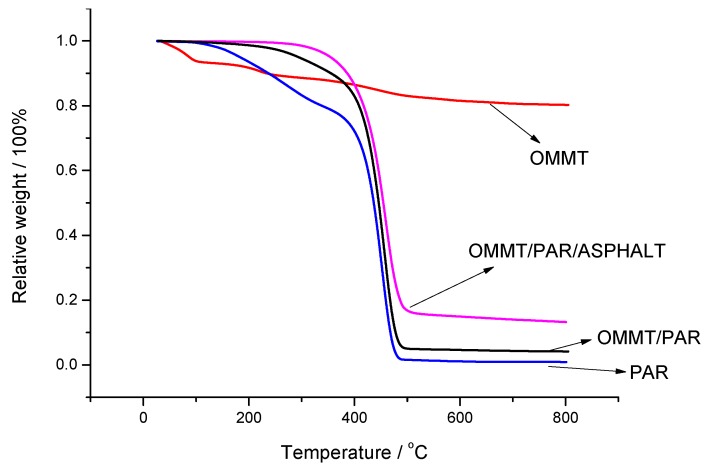
TG curves of OMMT, PAR and their composites.

Figure 4 and Table 7 show the confirmation that OMMT improves the thermal stability of the composites. From OMMT curve, it can be seen that there is a rapid reduction (weight loss of about 10%) at the beginning due to the decomposition of the organic compounds, and then it remains constant at 90%. This indicates that montmorillonoid is so strong that it will not decompose even under temperatures of 800 °C, showing amazing thermal stability. Once the OMMT/PAR is prepared, its decomposition temperature is enhanced to 293.6 °C, which is 111 °C higher that of PAR, which is 182.6 °C. Therefore, montmorillonoid can obviously increase thermal performance, even though only a small amount is added. Meanwhile, when OMMT/PAR is mixed with asphalt with content of only 3 wt %, the effcts on the thermal performance will also be visible, according to the curve of OMMT/PAR/ASPHALT. Hence, OMMT/PAR shows a dramatic effect on thermal stability according to Figure 2 and Figure 4. 

### 3.2. Adhesion Properties

**Figure 5:**
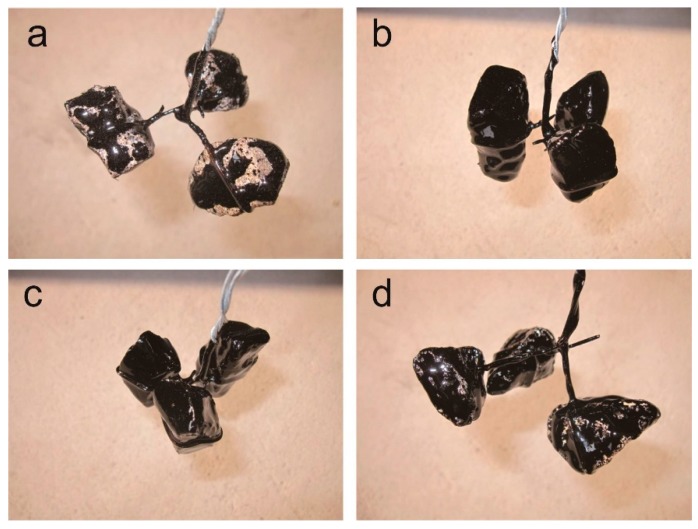
Adhesion test results between acidic aggregates and (**a**) matrix asphalt of SK-90#, (**b**) SK-90-OMMT/PAR, (**c**) SK-90-ASA-1 and (**d**) SK-90-ASA-2.

The results of the adhesion properties between the acidic aggregates and asphalts modified by anti-stripping composites are shown in Figure 5, Table 8 and Table 9. In order to evaluate the adhesive durability, it is much more significant to investigate interfacial adhesive properties between aggregates and aging asphalts. Therefore, both experimental aging methods—the rolling thin film oven test (RTFOT) and pressure aging vessel (PAV)—were employed, and the adhesion properties with aging modified asphalts were also tested. Accordingly, both of the KL-90# and SK-90# asphalts showed the same results, wherein the adhesion properties are poor (1 or 2 grade) between the matrix asphalts and the acidic aggregates. Once the asphalts had been modified by anti-stripping agents, the adhesion grade could be enhanced from 2 grade to the best grade (grade 5), when the asphalts had not undergone any aging processes, as indicated by “none” in Table 8 and Table 9. For the results of modified asphalts after RTFOT, the ASA-2-modified ones showed the lowest results, 3 grade, compared with those of OMMT/PAR and ASA-1, which were both 5 grade. Similarly, when the modified asphalts underwent PAV aging, OMMT/PAR-modified asphalts were still 5 grade, compared with the results for ASA-1- and ASA-2-modified asphalts, which were only 4 and 3 grade, respectively. 

In brief, all the asphalts modified by the anti-stripping products give a temporary upgrade when they have not undergone aging, but their adhesion properties are so different after RTFOT and PAV aging. Also, other acidic aggregates of sandstone and quartzite have shown similar results. That is to say, ASA-1 and ASA-2 cannot prevent decomposition throughout the entire cycle of use during aging processes due to their poor thermal stabilities, which cannot enhance the interfacial adhesive strengths constantly and are therefore highly unsatisfactory for the service life of the highway.

### 3.3. Properties of the Asphalts Modified by Anti-Stripping Composites

It can be seen in Table 10 that the penetrations, softening points and ductilities are changed to a slight degree among the modified asphalts. The viscosities in Table 11 are slightly enhanced following modification with anti-stripping composites. Different anti-stripping materials show similar effects on asphalt viscosity. The modified asphalt’s properties are closely related to the construction progress and quality, and the pitch viscosity at 135 °C has a remarkable effect on construction progress. That is to say, the mild viscosity enhancements are approximately equivalent with the addition of various different anti-stripping composites. This indicates that variations of asphalt viscosity are paralleled by using OMMT/PAR or other counterparts, which may affect the asphalt content in the next step of the asphalt mix design process.

Asphalts modified by anti-stripping materials exhibit no weight loss after RTFOT, based on Table 12, indicating that the asphalts are able to experimentally undergo short-term aging. Moreover, the residual penetration ratio after RTFOT aging must be more than 57% according to TSCHAP. Asphalt modified by OMMT/PAR increases to a maximum of 80%, compared with the other values of 72% (ASA-1) and 78% (ASA-2). Usually, the ductility of asphalt is shortened after RTFOT, so the larger the ductility is after RTFOT, the better the anti-cracking properties of the asphalt can be. For the residual ductility after RTFOT, asphalt modified by OMMT/PAR achieves the highest value of 24.3 cm, compared to those of 16.5 cm (ASA-1) and 18.2 cm (ASA-2).

PG grades after PAV can evaluate the application performances and limitations under both cold and hot environments. As shown in Table 12, the matrix asphalt is PG 70-22, while other asphalts modified by any anti-stripping composite changed uniformly to PG 64-28. It can be concluded that anti-stripping composites can help asphalts to be employed in much lower temperatures, from −22 °C to −28 °C, but the resilience to high temperatures is sadly also lowered, accordingly. By contrast, if it is necessary for the asphalts to be used in higher temperature areas, the matrix asphalts can be chosen that have lower needle penetration, or anti-rutting agent can be used. Therefore, the asphalt modified by OMMT/PAR shows the best properties compared to other agents.

### 3.4. Properties of Modified Asphalt Mixtures

**Figure 6:**
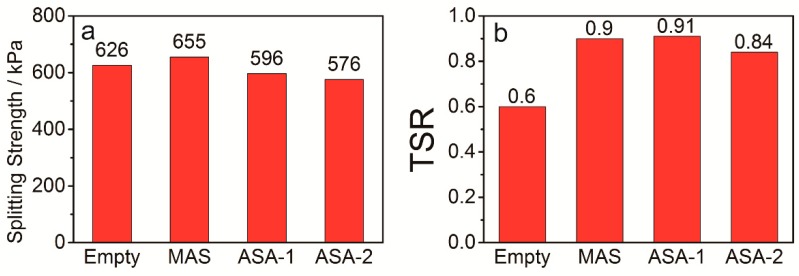
(**a**) Freeze–thaw splitting strength and (**b**) TSR of asphalt mixture, where Empty represents asphalt mixtures employed with matrix asphalts, and MAS, ASA-1 and ASA-2 are the asphalt mixtures prepared using the asphalts modified with MAS, ASA-1 and ASA-2, respectively.

Since splitting failure occurs when the asphalt mixture suffers from moisture damage, the freeze–thaw splitting test of asphalt mixture is used to measure the damage intensity ratio before and after moisture damage, so as to study the water stability of the asphalt mixture. Figure 6 and Table 13 show that splitting strength changes differentially, and TSR is obviously enhanced after the asphalts are modified with anti-stripping agents. The asphalt mixture prepared using MAS-modified asphalt presented the highest splitting strength of 655 kPa, and those prepared using ASA-1- and ASA-2-modified asphalts showed the lowest splitting strengths. It is supposed that ASA-1 and ASA-2 decomposed and lost their ability to prevent stripping. Based on TSR, asphalt mixtures prepared using matrix asphalt have the lowest value of 0.6, while those prepared using MAS- and ASA-1-modified asphalts presented the top values of 0.9 and 0.91, respectively. It can be concluded that asphalt mixtures with MAS can enhance the interfacial adhesive properties even though they exhibted poor performance in the the freeze–thaw cycle test, which also confirms the results of the thermal and adhesion analyses. 

Dynamic elasticity modulus simulates the dynamic effect condition of vehicles; the modulus is measured at a quick loading rate, and can be defined as the ratio between maximum stress and maximum reversible axial strain. The vast majority of pavement materials are viscous-elastic plastic complexes, and they are sensitive to time, temperature, loading conditions, etc. Therefore, it better approaches the actual pavement situation and is more practical than the static modulus of elasticity. Table 14 shows the dynamic elasticity modulus data and illustrates that the dynamic modulus of elasticity is increased by utilizing anti-stripping agents. Asphalt mixtures prepared using MAS-modified asphalt showed the best modulus compared with those prepared using ASA-1 and ASA-2, which showed the best pavement development of all. 

## 4. Adhesion Mechanism Analysis

**Figure 7:**
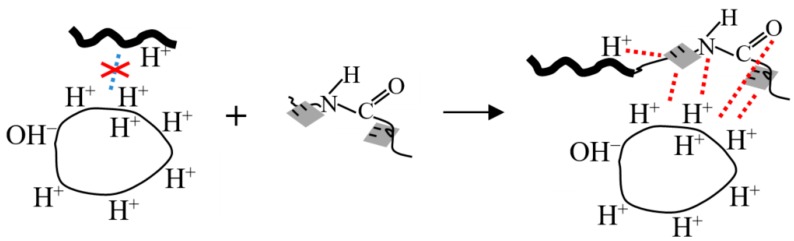
Schematic representation of interfacial adhesion mechanism between asphalt modified by OMMT/PAR and acidic aggregate.

The interfacial adhesion mechanism between the asphalt and acidic aggregate is shown in Figure 7. There are abundant hydrogen ions or groups on the molecular chain due to the specific asphalt manufacturing technique, thus asphalt is generally acidic. When asphalt mixes with an alkaline aggregate, a neutralization reaction occurs between the acidic group of the asphalt and the alkaline group of the aggregate. Furthermore, tight adhesion can be confirmed, and this is why asphalt possesses better adhesion capability with alkaline aggregates than acidic ones. In contrast, when asphalt and acidic aggregates are mixed, repulsion interactions take place between the acidic groups of the asphalt and the aggregate, with only partial interfaces (surfaces without or with a few hydrogen ions) performing adhesion in the mixing procedure, and asphalt envelops the aggregate only as a result of its viscidity on other surfaces. Once the asphalt mixtures have been placed in a moist environment, electronegative hydroxyls in water can easily enter into asphalt/acidic aggregate interfaces due to the attraction of positive charge, which causes moisture damage to the pavement.

For the asphalt mixture using acidic aggregates, their surfaces are wrapped with hydrogen ions from OMMT/PAR; the N and O in OMMT/PAR can combine with the H from the aggregates’ surface by means of a hydrogen bond. Furthermore, OMMT layers in OMMT/PAR have a parallel composition compared to aggregates, are negatively charged, and can be electrostatically attracted by a positive charge on the aggregates’ surface, according to electronic theory, and the surfaces are coated with OMMT/PAR molecules; H atoms present in asphalt can combine with OMMT/PAR through hydrogen bonds. According to the solubility parameter close principle, macromolecular asphalt completely adheres to aggregate coated with abundant macromolecular OMMT/PAR, and the adhesion between the acidic aggregate and the asphalt is dramatically improved, resulting in a better thermal stability at the interfaces.

## 5. Conclusions

According to our novel anti-stripping OMMT/PAR composite, the interfacial adhesion properties were improved compared to other products. The conclusions are as follows.

1. Asphalt anti-stripping OMMT/PAR composite modified by OMMT showed high decomposition temperature, and enhanced the thermal stability and effectively provided excellent interfacial adhesive properties, although it suffered under high-temperature environments in the mix plant or during transportation.
2. Asphalt modified by OMMT/PAR could reach the 5 grade, compared to the 4 grade (ASA-1) and 3 grade (ASA-2) reached after PAV, respectively, suggesting that asphalt modified by OMMT/PAR is able to keep its effects indefinitely, and shows excellent durability during aging.
3. Asphalt modified with OMMT/PAR and their mixture also showed excellent pavement properties compared with others. Moreover, the moisture damage process and the reasons for OMMT/PAR improving the interfacial adhesion properties were explained by adhesion mechanism analysis.

Based on the study above, OMMT/PAR anti-stripping composites show a successful improvement of the interfacial adhesive property between acidic aggregates and asphalt.

## Figures and Tables

**Table 1:** Physical properties of KL-90# asphalt.

Test Item	Requirements	Results	Test Basis
Penetration/0.1 mm (100 g, 5 s), 25 °C	80–100	84	T0604
Penetration index (PI)	−1.5 to +1.0	−1.01	T0604
Softening point (TR&B)/°C	≮44	46.5	T0606
Ductility (15 °C, 5 cm/min)/cm	≮100	>100	T0605
Flash point/°C	≮260	330	T0611
Solubility/%	≮99.5	99.7	T0607
Density (15 °C)/g/cm^3^	-	0.984	T0603
Mass loss/%	≯±0.4	−0.07	T0609
Residual penetration ratio (25 °C)/%	≮57	75	T0609
Residual ductility (10 °C)/cm	≮8	28	T0604

**Table 2:** Physical properties of SK-90# asphalt.

Test Item	Requirements	Results	Test Basis
Penetration/0.1 mm (100 g, 5 s), 25 °C	80–100	83	T0604
Penetration index (PI)	−1.5 to +1.0	−1.15	T0604
Softening point (TR&B)/°C	≮44	45.5	T0606
Ductility (15 °C, 5 cm/min)/cm	≮100	>100	T0605
Flash point/°C	≮260	326	T0611
Solubility/%	≮99.5	99.8	T0607
Density (15 °C)/g/cm^3^	-	1.035	T0603
Mass loss/%	≯±0.4	−0.08	T0609
Residual penetration ratio (25 °C)/%	≮57	64	T0609
Residual ductility (10 °C)/cm	≮8	8	T0604

**Table 3:** Physical properties of granite.

Test Item	Requirements	Results	Test Basis
Apparent relative density	≮2.5	2.822	T0304
Bulk volume relative density	-	2.800	T0304
Water absorption/%	≯3.0	0.5	T0304
Acicular content/%	≯20	6.6	T0312
<0.075 mm particle content/%	≯1.0	0.2	T0310

**Table 4:** Physical properties of sandstone.

Test Item	Requirements	Results	Test Basis
Apparent relative density	≮2.5	2.767	T0304
Bulk volume relative density	-	2.730	T0304
Water absorption/%	≯3.0	0.6	T0304
Acicular content/%	≯20	10.6	T0312
<0.075 mm particle content/%	≯1.0	0.4	T0310

**Table 5:** Physical properties of quartzite.

Test Item	Requirements	Results	Test Basis
Apparent relative density	≮2.5	2.756	T0304
Bulk volume relative density	-	2.690	T0304
Water absorption/%	≯3.0	0.8	T0304
Acicular content/%	≯20	13.4	T0312
<0.075 mm particle content/%	≯1.0	0.3	T0310

**Table 6:** Thermolysis temperature of anti-stripping composites of MAS, ASA-1 and ASA-2.

Anti-Stripping Composites	Thermolysis (2%) Temperature/°C	Thermolysis (5%) Temperature/°C
MAS	145	202
ASA-1	122	151
ASA-2	141	183

**Table 7:** Thermolysis temperature of OMMT, PAR and their composites.

Materials	Thermolysis (5%) Temperature/°C
OMMT	87.0
PAR	182.6
OMMT/PAR	293.6
Matrix asphalt	343.9
OMMT/PAR/ASPHALT	354.2

**Table 8:** Adhesion grades of KL-90 asphalt modified by anti-stripping composites and granite.

Asphalt Kinds	Aging Methods	Adhesion Grades
Matrix asphalt KL-90	none	2
	RTFOT	1
	PAV	1
KL-90-OMMT/PAR	none	5
	RTFOT	5
	PAV	5
KL-90-ASA-1	none	5
	RTFOT	5
	PAV	4
KL-90-ASA-2	none	5
	RTFOT	3
	PAV	3

**Table 9:** Adhesion grades of SK-90 asphalt modified by anti-stripping composites and granite.

Asphalt Kinds	Aging Methods	Adhesion Grades
Matrix asphalt SK-90	none	2
	RTFOT	1
	PAV	1
SK-90-OMMT/PAR	none	5
	RTFOT	5
	PAV	5
SK-90-ASA-1	none	5
	RTFOT	5
	PAV	4
SK-90-ASA-2	none	5
	RTFOT	3
	PAV	3

**Table 10:** Three indices of asphalts modified by anti-stripping composite.

Items	KL-90	KL-90-OMMT/PAR	KL-90-ASA-1	KL-90-ASA-2
Penetration/0.1 mm (100 g, 5 s), 25 °C	94	80	85	79
Softening point (TR&B)/°C	47.5	50	48.5	48.5
Ductility (15 °C, 5 cm/min)/cm	>100	>100	>100	>100

**Table 11:** Viscosity indices of asphalts with anti-stripping composites.

Items	KL-90	KL-90-OMMT/PAR	KL-90-ASA-1	KL-90-ASA-2
60 °C Viscosity/mPa·s	279	220	300	270
135 °C Viscosity/mPa·s	0.59	0.882	0.895	0.865
175 °C Viscosity/mPa·s	-	0.630	0.640	0.645

**Table 12:** Aging indices of asphalts with anti-stripping composites.

Items	KL-90	KL-90-OMMT/PAR	KL-90-ASA-1	KL-90-ASA-2
OMMT/PARs loss after RTFOT/%	0	0	0	0
Residual penetration ratio after RTFOT (25 °C)/%	70	80	72	78
Residual ductility after RTFOT (10 °C)/cm	18	24.3	16.5	18.2
PG grades after PAV	70, −22	64, −28	64, −28	64, −28

**Table 13:** Freeze–thaw splitting strength and strength ratio of asphalt mixtures.

Modified Materials	Freeze–Thaw Splitting Strength	TSR
Empty (Matrix asphalt)	626 kPa	0.60
MAS	655 kPa	0.90
ASA-1	596 kPa	0.91
ASA-2	576 kPa	0.84

**Table 14:** Dynamic elasticity modulus of asphalt mixtures with anti-stripping composites.

Asphalt	Dynamic Elasticity Modulus/MPa
Matrix asphalt of KL-90	4894
KL-90-OMMT/PAR	5257
KL-90-ASA-1	5085
KL-90-ASA-2	4920
